# Supplementary material for: Effects of Higher Normal Blood Pressure on Brain Are Detectable before Middle-Age and Differ by Sex
Source: J Clin Med. 2022 May 31;11(11):3127. doi: 10.3390/jcm11113127 (PMC9181456; doi:10.3390/jcm11113127)
Supplement: Supplementary file 1 [file jcm-11-03127-s001.zip › jcm-1681564-supplementary.pdf]

Supplement

Title: Effects of higher normal blood pressure on brain are detectable before middle-age and differ by sex.

Khawlah Alateeq, Centre for Research on Ageing, Health and Wellbeing, 54 Mills Road, Australian National University, Canberra, ACT 2601, Australia.
Email: Khawlah.alateeq@anu.edu.au

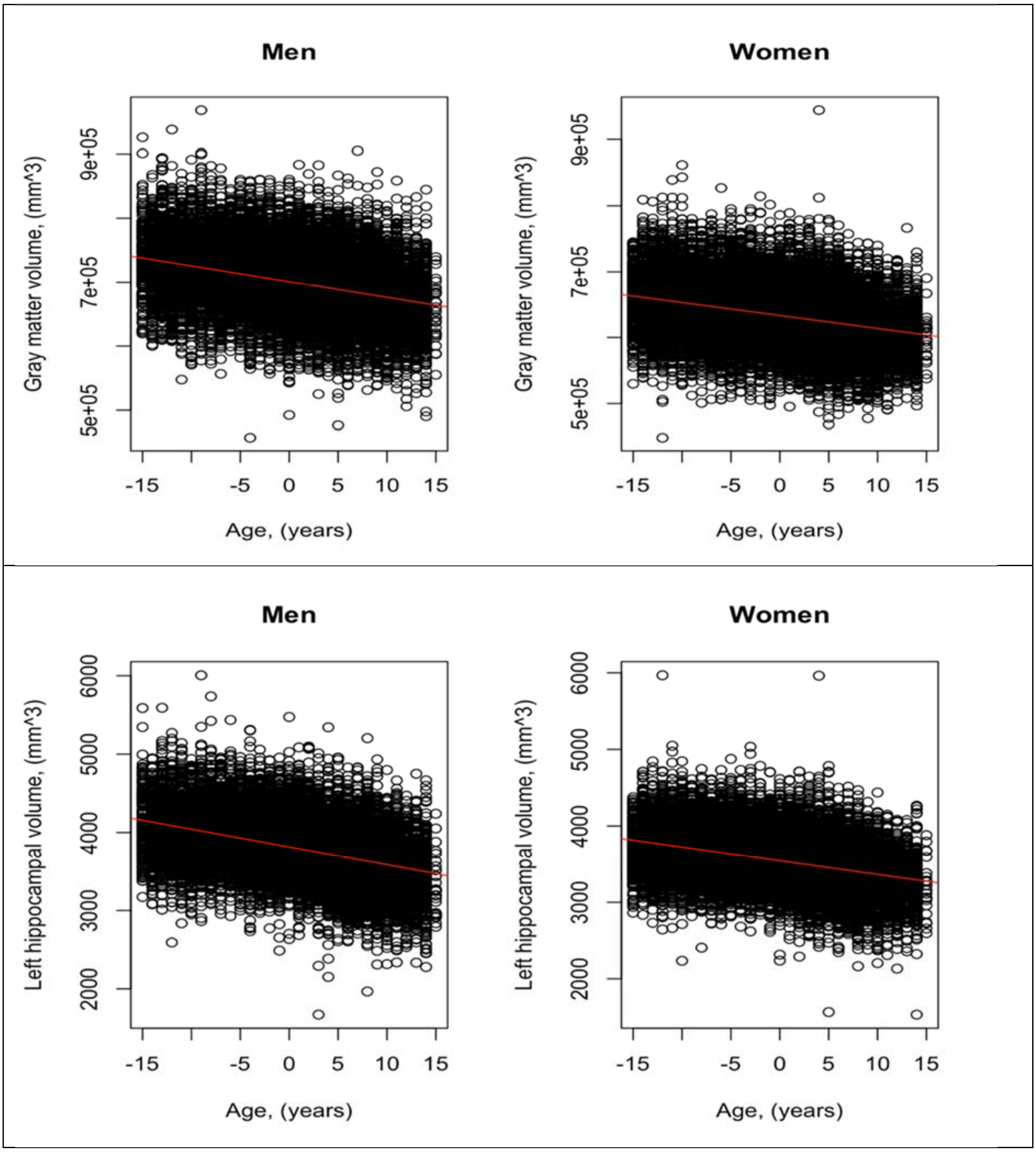

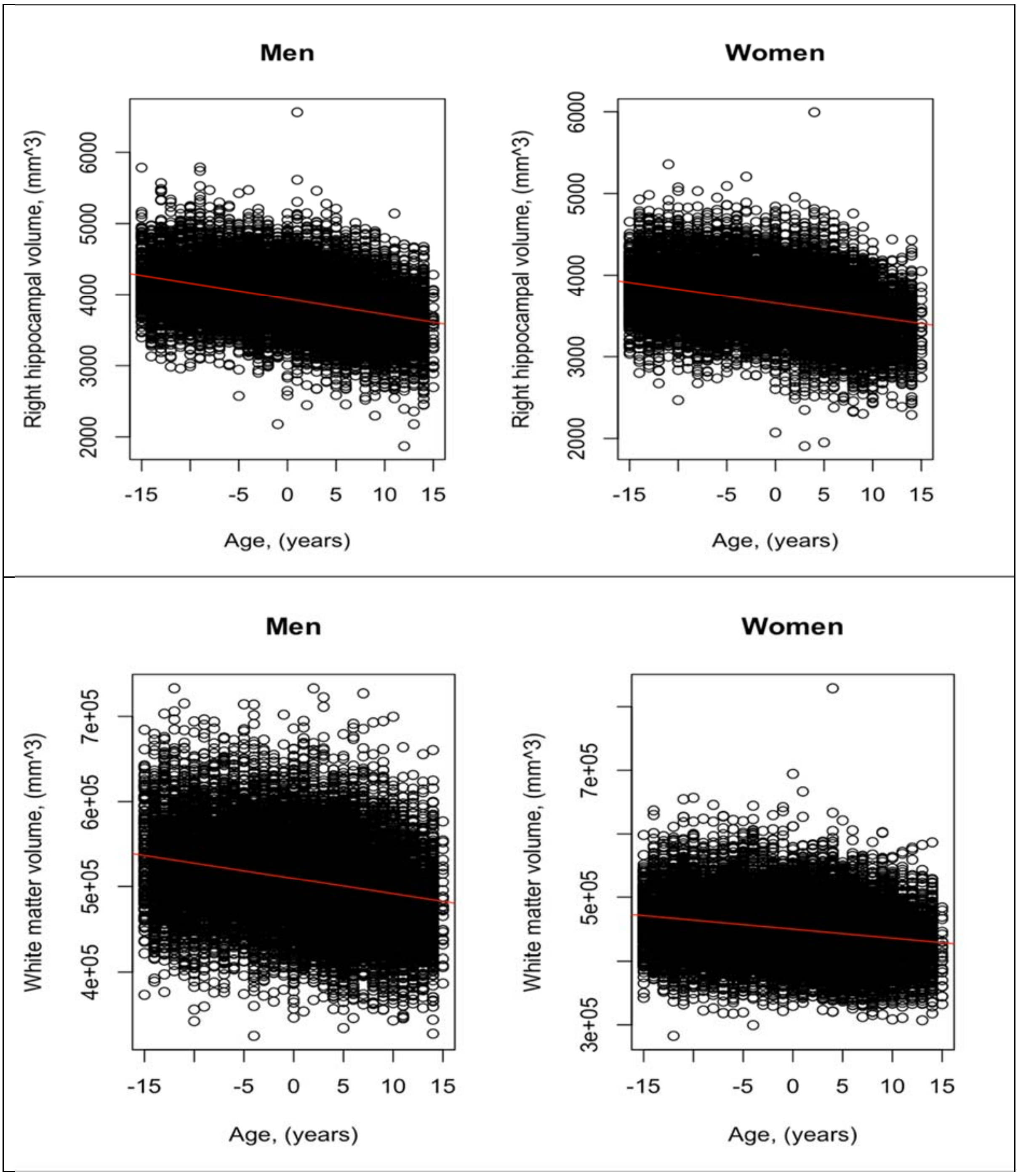

Figure S1. Scattered plot shows the distribution of the brain volumes across age in men and women.

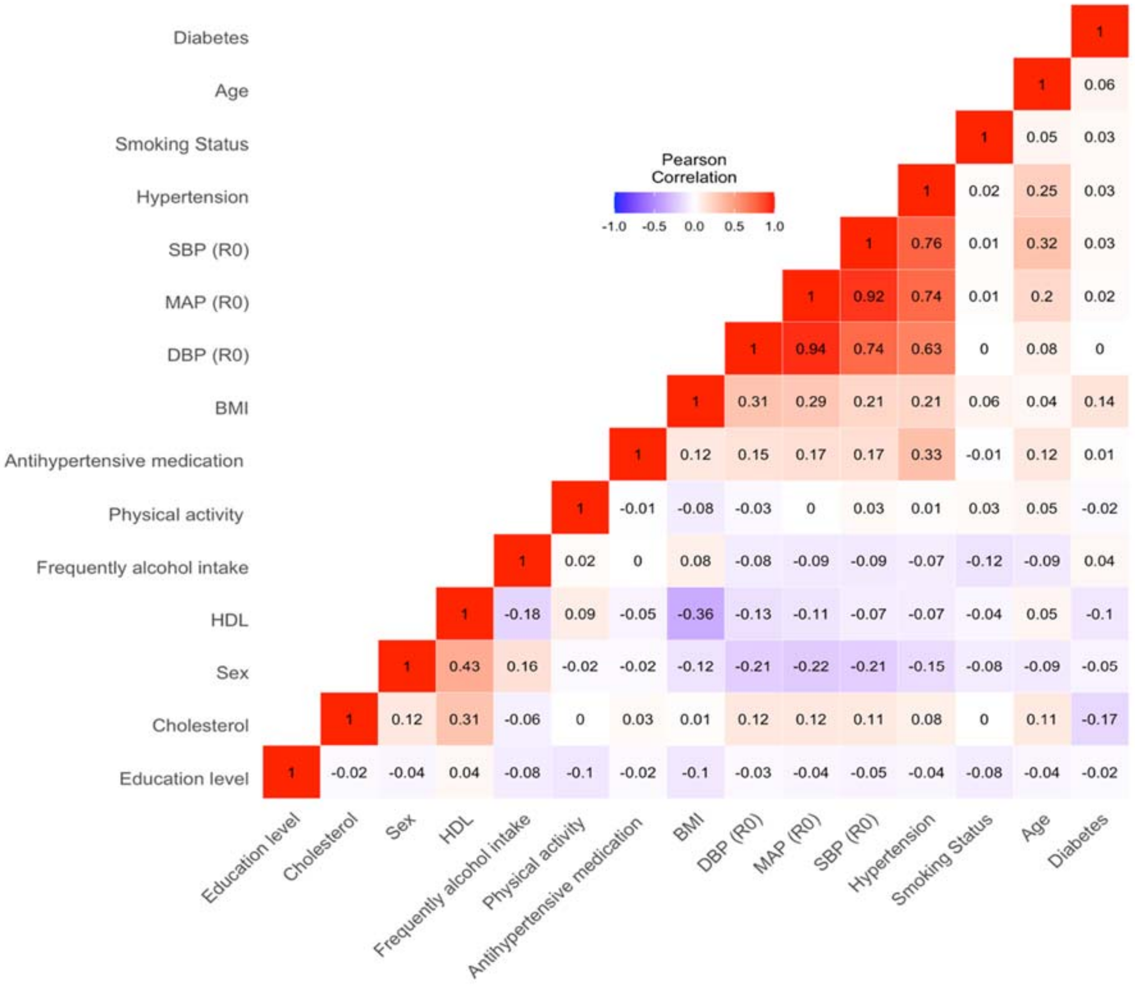

Figure S2. Bivariate correlation matrix between BP measurements and main covariates

Table S1. Interaction between BP (MAP, SBP, DBP), antihypertensive medication, and brain volumes across age groups in men and women at UK biobank study.

	**Gray Matter volume (mm^3)**		**White matter volume (mm^3)**		**Left Hippocampus volume (mm^3)**		**Right Hippocampus volume (mm^3)**		**White matter lesions volume (mm^3)**	
	Beta (SE)		Beta (SE)		Beta (SE)		Beta (SE)		Beta (SE)	
	**Participants aged ≤45 years**									
	Men, n=2220	Women, n=2867	Men, n=2220	Women, n=2867	Men, n=2220	Women, n=2867	Men, n=2220	Women, n=2867	Men, n=2220	Women, n=2867
MAP	-253.756*** (63.218)	-239.306*** (48.302)	4.574 (64.455)	-121.733** (48.375)	1.015 (0.677)	-1.206** (0.526)	0.729 (0.685)	-1.154** (0.522)	0.003*** (0.001)	0.003*** (0.001)
Antihypertensive medication	10,736.320 (8,364.695)	6,953.476 (5,730.181)	-6,220.868 (8,528.338)	-7,673.860 (5,738.851)	89.592 (89.528)	-24.431 (62.348)	111.500 (90.686)	-59.839 (61.983)	0.184* (0.108)	0.072 (0.083)
MAPX antihypertensive medication	-582.093 (387.614)	-269.292 (294.586)	110.401 (395.197)	429.391 (295.032)	-6.599 (4.149)	1.888 (3.205)	-5.706 (4.202)	3.684 (3.187)	-0.004 (0.005)	-0.003 (0.004)
SBP	-167.489*** (48.291)	-168.342*** (35.741)	-0.293 (49.182)	-92.447*** (35.781)	0.946* (0.516)	-0.672* (0.389)	0.971* (0.523)	-0.590 (0.387)	0.002*** (0.001)	0.002*** (0.001)
Antihypertensive medication	17,605.070 (10,811.920)	5,772.999 (5,736.367)	9,034.142 (11,011.450)	-5,983.785 (5,742.761)	231.027** (115.522)	-31.935 (62.409)	229.638** (117.014)	-72.332 (62.044)	0.198 (0.140)	0.111 (0.083)
SBPX antihypertensive medication	-632.666* (343.978)	-143.318 (197.242)	-445.209 (350.326)	208.438 (197.462)	-9.147** (3.675)	1.389 (2.146)	-7.882** (3.723)	2.779 (2.133)	-0.003 (0.004)	-0.004 (0.003)
DBP	-272.067*** (67.772)	-251.103*** (53.464)	8.383 (69.063)	-119.825** (53.525)	0.818 (0.726)	-1.463** (0.581)	0.300 (0.735)	-1.459** (0.578)	0.003*** (0.001)	0.003*** (0.001)
Antihypertensive medication	7,226.912 (7,460.247)	7,268.042 (6,044.473)	-11,410.250 (7,602.306)	-9,553.892 (6,051.337)	31.639 (79.870)	-19.289 (65.724)	62.570 (80.896)	-48.780 (65.339)	0.177* (0.097)	0.041 (0.088)
DBPX	-465.765 (385.695)	-334.602 (350.222)	439.600 (393.039)	589.543* (350.620)	-3.974 (4.129)	1.756 (3.808)	-3.370 (4.182)	3.334 (3.786)	-0.004 (0.005)	-0.001 (0.005)

antihypertensive medication										
**Participants aged between 46-55 years**										
	Men, n=5699	Women, n=7456	Men, n=5699	Women, n=7456	Men, n=5699	Women, n=7456	Men, n=5699	Women, n=7456	Men, n=5699	Women, n=7456
MAP	-221.478*** (39.579)	-225.116*** (27.107)	-119.315*** (38.578)	-61.891** (27.997)	-0.538 (0.405)	-0.249 (0.291)	-0.416 (0.409)	-0.368 (0.288)	0.006*** (0.001)	0.007*** (0.001)
Antihypertensive medication	-9,138.201*** (3,446.335)	-2,998.427 (2,326.933)	-7,549.108** (3,359.214)	-1,829.803 (2,403.383)	-77.124** (35.233)	-52.064** (25.013)	-92.939*** (35.648)	-42.420* (24.739)	0.099* (0.053)	0.074* (0.044)
MAPX antihypertensive medication	174.165 (172.530)	-71.032 (122.745)	65.078 (168.168)	20.968 (126.778)	1.674 (1.764)	0.265 (1.319)	2.443 (1.785)	-0.003 (1.305)	0.001 (0.003)	0.002 (0.002)
SBP	-141.922*** (28.579)	-148.837*** (18.582)	-87.669*** (27.839)	-8.915 (19.192)	-0.153 (0.292)	-0.064 (0.200)	-0.130 (0.295)	-0.086 (0.198)	0.004*** (0.0004)	0.005*** (0.0003)
Antihypertensive medication	-10,520.590*** (3,791.735)	-3,063.157 (2,550.758)	-8,700.389** (3,693.626)	-2,089.413 (2,634.558)	-93.992** (38.743)	-50.036* (27.412)	-116.641*** (39.195)	-44.898* (27.113)	0.106* (0.058)	0.080* (0.048)
SBPX antihypertensive medication	144.948 (120.108)	-45.338 (81.446)	77.874 (117.001)	4.081 (84.122)	1.540 (1.227)	0.022 (0.875)	2.276* (1.242)	0.001 (0.866)	0.001 (0.002)	0.001 (0.002)
DBP	-245.404**** (44.291)	-243.326**** (31.612)	-118.727*** (43.176)	-113.206**** (32.610)	-0.829* (0.453)	-0.415 (0.340)	-0.626 (0.458)	-0.626* (0.336)	0.006**** (0.001)	0.008**** (0.001)
Antihypertensive medication	-8,350.592** (3,260.427)	-3,789.582* (2,200.205)	-6,946.588** (3,178.400)	-1,709.401 (2,269.659)	-63.223* (33.327)	-53.449** (23.632)	-72.957** (33.725)	-41.396* (23.371)	0.110** (0.050)	0.094** (0.041)
DBPX antihypertensive medication	153.368 (192.681)	-48.831 (140.824)	30.380 (187.834)	39.076 (145.270)	1.153 (1.970)	0.506 (1.513)	1.637 (1.993)	0.018 (1.496)	0.001 (0.003)	0.001 (0.003)
**Participants aged between 56-65 years**										
	Men, n=7708	Women, n=7692	Men, n=7708	Women, n=7692	Men, n=7708	Women, n=7692	Men, n=7708	Women, n=7692	Men, n=7708	Women, n=7692

MAP	-179.243*** (34.484)	-170.099*** (29.764)	-74.477** (34.563)	-89.883*** (30.146)	-0.230 (0.364)	-0.292 (0.318)	0.031 (0.371)	-0.447 (0.320)	0.009*** (0.001)	0.009*** (0.001)
Antihypertensive medication	-5,871.521** (2,368.242)	-5,915.991*** (1,842.628)	-6,716.384*** (2,373.675)	-2,567.880 (1,866.239)	-39.313 (24.995)	-3.128 (19.717)	-44.987* (25.445)	4.338 (19.781)	0.245*** (0.046)	0.228*** (0.042)
MAPX antihypertensive medication	65.252 (120.682)	117.197 (102.060)	192.206 (120.958)	-51.427 (103.368)	0.559 (1.274)	-1.393 (1.092)	0.630 (1.297)	-1.342 (1.096)	-0.006*** (0.002)	-0.004* (0.002)
SBP	-145.078*** (22.678)	-102.494*** (18.682)	-51.153** (22.750)	-59.992*** (18.918)	-0.340 (0.240)	-0.243 (0.200)	-0.091 (0.244)	-0.291 (0.200)	0.006*** (0.0004)	0.006*** (0.0004)
Antihypertensive medication	-7,597.765*** (2,701.247)	-5,736.028*** (2,141.589)	-6,389.681** (2,709.862)	-2,741.243 (2,168.619)	-50.945* (28.532)	9.122 (22.910)	-54.738* (29.048)	18.927 (22.984)	0.253*** (0.053)	0.242*** (0.049)
SBPX antihypertensive medication	91.287 (76.925)	48.198 (62.436)	92.552 (77.171)	-20.251 (63.225)	0.697 (0.813)	-1.082 (0.668)	0.667 (0.827)	-1.151* (0.670)	-0.003** (0.002)	-0.002 (0.001)
DBP	-139.146**** (40.429)	-182.269**** (35.954)	-72.275* (40.485)	-85.518** (36.407)	0.065 (0.426)	-0.189 (0.385)	0.208 (0.434)	-0.438 (0.386)	0.008**** (0.001)	0.010**** (0.001)
Antihypertensive medication	-5,014.314** (2,082.230)	-6,073.270**** (1,560.044)	-6,453.186*** (2,085.096)	-2,985.786* (1,579.697)	-31.034 (21.956)	-17.182 (16.688)	-36.877* (22.350)	-11.228 (16.743)	0.238**** (0.041)	0.233**** (0.036)
DBPX antihypertensive medication	-1.233 (140.241)	173.790 (117.737)	241.160* (140.434)	-51.779 (119.220)	0.006 (1.479)	-0.797 (1.259)	0.170 (1.505)	-0.545 (1.264)	-0.007*** (0.003)	-0.006** (0.003)
**Participants aged >65 years**										
	Men, n=1541	Women, n=1077	Men, n=1541	Women, n=1077	Men, n=1541	Women, n=1077	Men, n=1541	Women, n=1077	Men, n=1541	Women, n=1077
MAP	-155.441* (79.747)	-148.297* (77.770)	-70.501 (77.678)	26.134 (81.717)	-1.174 (0.807)	1.592* (0.849)	-1.680** (0.839)	1.073 (0.822)	0.009*** (0.002)	0.010*** (0.002)
Antihypertensive medication	-2,411.661 (4,986.035)	-4,363.604 (4,150.917)	782.638 (4,856.624)	-514.460 (4,361.551)	-40.734 (50.458)	-18.131 (45.288)	-70.617 (52.472)	-26.950 (43.860)	0.227** (0.107)	0.331*** (0.111)

MAPX antihypertensive medication	257.642 (245.861)	52.635 (229.188)	141.962 (239.480)	-27.067 (240.818)	3.399 (2.488)	-0.971 (2.501)	5.161** (2.587)	0.905 (2.422)	-0.0003 (0.005)	-0.013** (0.006)
SBP	-55.179 (49.730)	-51.515 (48.786)	-7.678 (48.415)	16.477 (51.191)	-0.016 (0.503)	1.184** (0.531)	-0.427 (0.523)	0.735 (0.515)	0.005*** (0.001)	0.006*** (0.001)
Antihypertensive medication	-3,290.270 (5,946.494)	-4,518.298 (5,231.465)	1,421.212 (5,789.182)	2,135.564 (5,489.312)	-75.655 (60.131)	-2.529 (56.968)	-91.064 (62.581)	-25.431 (55.203)	0.330*** (0.127)	0.434*** (0.140)
SBPX antihypertensive medication	139.577 (148.142)	18.345 (139.363)	43.781 (144.223)	-91.102 (146.232)	2.457 (1.498)	-0.932 (1.518)	2.917* (1.559)	0.337 (1.471)	-0.003 (0.003)	-0.009** (0.004)
DBP	-233.480** (95.639)	-224.696** (92.970)	-138.032 (93.176)	26.304 (97.781)	-2.508*** (0.967)	1.260 (1.016)	-2.837*** (1.006)	0.964 (0.984)	0.009**** (0.002)	0.012**** (0.002)
Antihypertensive medication	-813.451 (3,996.848)	-4,193.422 (3,282.286)	1,119.898 (3,893.926)	-1,779.415 (3,452.160)	-4.091 (40.425)	-25.752 (35.883)	-34.069 (42.053)	-20.926 (34.733)	0.191** (0.086)	0.240*** (0.088)
DBPX antihypertensive medication	282.305 (289.778)	83.703 (273.102)	216.338 (282.316)	117.245 (287.237)	2.411 (2.931)	-0.423 (2.986)	5.261* (3.049)	1.174 (2.890)	0.004 (0.006)	-0.012* (0.007)

.* p<0.05; ** p<0.01; *** p<0.001

Note: Hierarchical linear regression models were used to quantify the correlation (Beta, and SE, standard error) between mean arterial pressure; MAP and brain volumes, while adjusting for basic covariates: total intracranial volume, and main covariates: HDL, cholesterol, diabetes, smoking status, higher education, physical activity, alcohol intake, antihypertensive medication and tested for the statistical interactions between (BP × antihypertensive medication) in predicting brain volume measures in model 2. Note: for ease of interpretation, we exponentiated the coefficients to produce the proportionate effect of a 10 mm Hg higher blood pressure on mean brain volume for each brain region in men and women. For instance, an exponentiated coefficient of 1.1 reflects a 10% increase in mean brain volume.

Table S2. Association between baseline adjusted SBP by PP and brain volumes across age groups in men and women at UK biobank study.

	Grey Matter volume (mm^3)		White matter volume (mm^3)		Left Hippocampus volume (mm^3)		Right Hippocampus volume (mm^3)		White matter lesions volume (mm^3)	
	Beta (SE)		Beta (SE)		Beta (SE)		Beta (SE)		Beta (SE)	
**Participants aged ≤ 45 years**										
	Men, n=2220	Women, n=2867	Men, n=2220	Women, n=2867	Men, n=2220	Women, n=2867	Men, n=2220	Women, n=2867	Men, n=2220	Women, n=2867
Non adjusted SBP	-179.415*** (47.880)	-172.891*** (35.186)	-8.685 (48.744)	-85.832** (35.229)	0.773 (0.512)	-0.628 (0.383)	0.823 (0.518)	-0.502 (0.381)	0.002*** (0.001)	0.002*** (0.001)
Adjusted SBP	-281.000*** (67.193)	-238.224*** (54.218)	25.600 (68.470)	-91.508* (54.307)	0.619 (0.719)	-1.497** (0.590)	0.046 (0.728)	-1.519*** (0.586)	0.003*** (0.001)	0.003*** (0.001)
PP	-83.378 (76.041)	-154.546*** (57.156)	-48.851 (77.183)	-100.699* (57.086)	1.036 (0.811)	0.005 (0.620)	1.803** (0.820)	0.292 (0.617)	0.002* (0.001)	0.001 (0.001)
**Participants aged between 46-55 years**										
	Men, n=5699	Women, n=7456	Men, n=5699	Women, n=7456	Men, n=5699	Women, n=7456	Men, n=5699	Women, n=7456	Men, n=5699	Women, n=7456
Non adjusted SBP	-133.847*** (27.786)	-151.197*** (18.091)	-83.331*** (27.064)	-8.702 (18.685)	-0.067 (0.284)	-0.062 (0.194)	-0.003 (0.287)	-0.086 (0.192)	0.004*** (0.0004)	0.005*** (0.0003)
Adjusted SBP	-229.585*** (44.219)	-213.428*** (32.223)	-103.752** (43.099)	-146.406*** (33.237)	-0.945** (0.452)	-0.486 (0.346)	-0.690 (0.457)	-0.791** (0.342)	0.006*** (0.001)	0.007*** (0.001)
PP	-86.948** (43.488)	-154.736**** (27.663)	-85.156** (42.308)	68.952** (28.487)	0.614 (0.444)	0.167 (0.297)	0.540 (0.449)	0.300 (0.293)	0.003**** (0.001)	0.005**** (0.001)
**Participants aged between 56-65 years**										
	Men, n=7708	Women, n=7692	Men, n=7708	Women, n=7692	Men, n=7708	Women, n=7692	Men, n=7708	Women, n=7692	Men, n=7708	Women, n=7692
Non adjusted SBP	-137.254*** (21.699)	-98.180*** (17.826)	-43.220** (21.768)	-61.805*** (18.050)	-0.280 (0.229)	-0.339* (0.191)	-0.034 (0.233)	-0.394** (0.191)	0.005*** (0.0004)	0.006*** (0.0004)

Adjusted SBP	-90.754** (39.736)	-141.865*** (35.175)	-39.446 (39.868)	-71.873** (35.622)	0.249 (0.420)	-0.109 (0.376)	0.296 (0.427)	-0.352 (0.378)	0.006*** (0.001)	0.008*** (0.001)
PP	-185.065**** (30.536)	-96.567**** (24.055)	-52.833* (30.629)	-67.790*** (24.341)	-0.594* (0.322)	-0.487* (0.257)	-0.205 (0.328)	-0.476* (0.258)	0.006**** (0.001)	0.006**** (0.001)
**Participants aged > 65 years**										
	Men, n=1541	Women, n=1077	Men, n=1541	Women, n=1077	Men, n=1541	Women, n=1077	Men, n=1541	Women, n=1077	Men, n=1541	Women, n=1077
Non adjusted SBP	-39.328 (46.797)	-49.280 (45.713)	-2.706 (45.547)	5.374 (47.974)	0.263 (0.473)	1.070** (0.498)	-0.096 (0.493)	0.776 (0.482)	0.005*** (0.001)	0.005*** (0.001)
Adjusted SBP	-219.645** (92.221)	-230.294** (89.603)	-134.598 (89.824)	44.540 (94.270)	-2.811*** (0.930)	0.850 (0.978)	-2.639*** (0.970)	0.879 (0.948)	0.009*** (0.002)	0.010*** (0.002)
PP	26.177 (61.855)	16.100 (60.908)	48.836 (60.179)	-9.546 (63.888)	1.509** (0.625)	1.315** (0.663)	0.892 (0.651)	0.849 (0.643)	0.004*** (0.001)	0.003* (0.002)

.* p<0.05; ** p<0.01; *** p<0.001

Note: Abbreviations: MAP, mean arterial pressure; SBP, systolic blood pressure; DBP, diastolic blood pressure. Model 1 was adjusted for total intracranial volume, and main covariates: HDL, cholesterol, diabetes, smoking status, higher education, physical activity, alcohol intake, and antihypertensive medication. Note: for ease of interpretation, we exponentiated the coefficients to produce the proportionate effect of a 10 mm Hg higher blood pressure on mean brain volume for each brain region in men and women. For instance, an exponentiated coefficient of 1.1 reflects a 10% increase in mean brain volume.
